# Synchronization of intrinsic 0.1‐Hz blood‐oxygen‐level‐dependent oscillations in amygdala and prefrontal cortex in subjects with increased state anxiety

**DOI:** 10.1111/ejn.13845

**Published:** 2018-02-08

**Authors:** Gert Pfurtscheller, Andreas Schwerdtfeger, Annemarie Seither‐Preisler, Clemens Brunner, Christoph Stefan Aigner, João Calisto, João Gens, Alexandre Andrade

**Affiliations:** ^1^ Institute of Neural Engineering Graz University of Technology Graz Austria; ^2^ BioTechMed Graz Graz Austria; ^3^ Institute of Psychology University of Graz 8010 Graz Austria; ^4^ Health Psychology and Applied Diagnostics University of Wuppertal Wuppertal Germany; ^5^ Department of Neuroradiology and Neurology University of Heidelberg Medical School Heidelberg Germany; ^6^ Centre for Systematic Musicology University of Graz Graz Austria; ^7^ Institute of Medical Engineering Graz University of Technology Graz Austria; ^8^ Institute of Biophysics and Biomedical Engineering Faculty of Sciences University of Lisbon Lisbon Portugal

**Keywords:** 0.1‐Hz oscillations, amygdala, blood‐oxygen‐level‐dependent signal, heart rate variability, state anxiety

## Abstract

Low‐frequency oscillations with a dominant frequency at 0.1 Hz are one of the most influential intrinsic blood‐oxygen‐level‐dependent (BOLD) signals. This raises the question if vascular BOLD oscillations (originating from blood flow in the brain) and intrinsic slow neural activity fluctuations (neural BOLD oscillations) can be differentiated. In this study, we report on two different approaches: first, on computing the phase‐locking value in the frequency band 0.07–0.13 Hz between heart beat‐to‐beat interval (RRI) and BOLD oscillations and second, between multiple BOLD oscillations (functional connectivity) in four resting states in 23 scanner‐naïve, anxious healthy subjects. The first method revealed that vascular 0.1‐Hz BOLD oscillations preceded those in RRI signals by 1.7 ± 0.6 s and neural BOLD oscillations lagged RRI oscillations by 0.8 ± 0.5 s. Together, vascular BOLD oscillations preceded neural BOLD oscillations by ~90° or ~2.5 s. To verify this discrimination, connectivity patterns of neural and vascular 0.1‐Hz BOLD oscillations were compared in 26 regions involved in processing of emotions. Neural BOLD oscillations revealed significant phase‐coupling between amygdala and medial frontal cortex, while vascular BOLD oscillations showed highly significant phase‐coupling between amygdala and multiple regions in the supply areas of the anterior and medial cerebral arteries. This suggests that not only slow neural and vascular BOLD oscillations can be dissociated but also that two strategies may exist to optimize regulation of anxiety, that is increased functional connectivity between amygdala and medial frontal cortex, and increased cerebral blood flow in amygdala and related structures.

## Introduction

Neural and hemodynamic oscillations in the slow and ultraslow frequency range between 0.01 and 0.2 Hz are of increasing interest because of their correlation with psychophysical performance and motor behavior (Vanhatalo *et al*., 2004; Fox *et al*., 2007; Monto *et al*., 2008; Palva & Palva, 2012; Pfurtscheller *et al*., 2017a). Fluctuations in electroencephalographic (EEG) and blood‐oxygen‐level‐dependent (BOLD) signals, which were investigated in detail by Palva & Palva (2012), provide converging evidence that oscillations between 0.01 and 0.2 Hz reflect quasi‐periodic excitability fluctuations in cortical and subcortical networks. EEG changes in the slow frequency range are closely associated with slow fluctuations in human psychophysical performance (Monto *et al*., 2008). Slow BOLD fluctuations (< 0.1 Hz) in the somatomotor cortex are correlated with behavioral fluctuations in motor performance, a finding which was termed the ‘BOLD‐behavior effect’ (Fox *et al*., 2007).

In contrast, widespread physiological low‐frequency oscillations in the frequency range between 0.01 and 0.2 Hz were identified as the predominant source of physiological noise in fMRI BOLD signals (Tong & Frederick, 2014). This raises the question as to whether some BOLD oscillations with a dominant frequency at 0.1 Hz have a neural origin. Due to neurovascular coupling, neural activation is followed by a hemodynamic change with a delay between 1.5 and 3.5 s (Golanov *et al*., 1994; Fox *et al*., 2007; Bruyns‐Haylett *et al*., 2013; Mateo *et al*., 2017). It is therefore expected that slow rhythmic neural activations are followed by hemodynamic oscillations. Golanov *et al*. (1994) reported a high correlation of 0.94 between the number of electrocorticographic bursts and regional cerebral blood flow waves per minute in the range of 0.07 to 0.14 Hz. Pfurtscheller *et al*. (2012a) reported on short‐time epochs (length about 100 s) of slow EEG alpha and/or beta power oscillations in sensorimotor areas followed by prefrontal oxyhemoglobin oscillations with frequencies of approximately 0.1 Hz. Noteworthy, beta power and gamma power oscillations with a dominant frequency at 0.1 Hz were reported at multiple electrodes placed in human posteromedial cortex (Foster & Parvizi, 2012).

In a recently published BOLD study, it was discussed the first time, whether intrinsic neural BOLD oscillations with a dominant frequency at 0.1 Hz may have their origin in the midcingulum (Pfurtscheller *et al*., 2017a). The method introduced is the measuring of phase‐coupling (PLV) and hence the time delay between 0.1‐Hz oscillations of heart beat‐to‐beat interval (RRI) and 0.1‐Hz BOLD oscillations. A positive time delay (pTD), indicating that RRI oscillations are leading, characterizes a neural BOLD signal, whereas a negative time delay (nTD), indicating that RRI oscillations are trailing, characterizes a vascular BOLD signal. The analysis of two resting states in 23 subjects revealed neural BOLD oscillations with a dominant frequency at 0.1 Hz in about 50% of the subjects.

In this study, we examined (i) whether neural 0.1‐Hz BOLD oscillations are found in the majority of subjects when more than two resting states are analyzed and (ii) whether a connectivity analysis of 0.1‐Hz BOLD oscillations between amygdala and prefrontal cortex, areas are known to be activated during processing of negative emotions (Phelps *et al*., 2001; Urry *et al*., 2009; Veer *et al*., 2011; Morawetz *et al*., 2017), can discriminate between neural and vascular BOLD oscillations. We hypothesized that slow neural BOLD oscillations are found in the majority of subjects and that a connectivity analysis of BOLD oscillations between amygdala and prefrontal regions is suitable to dissociate between neural and vascular BOLD oscillations. To test the hypothesis, we studied not only the coupling between RRI time courses and BOLD signals in four resting states recorded on the same day in 23 scanner‐naïve anxious subjects but calculated also the connectivity of 0.1‐Hz BOLD oscillations between 26 regions of interest (ROIs) including prefrontal regions, amygdala, cingulum and precuneus. We also assessed state anxiety in each resting state, because it is known that scanner participation is an anxiety‐provoking experience (Katz *et al*., 1994).

## Materials and Methods

### Experimental paradigm and within‐scanner questionnaire

A total of 25 participants (12 female, 22 right‐handed) between 19 and 34 years (mean ± SD: 24 ± 3.2 years) took part in the study. Two participants were excluded from further analysis due to cardiac arrhythmia. All participants were naïve to the purpose of the study, had no former MRI experience, had normal or corrected‐to‐normal vision and were without any record of neurological or psychiatric disorders (as assessed by self‐report). All participants gave informed written consent to the protocol of the study, which had been approved by the local Ethics Committee at the University of Graz.

The experimental task consisted of four resting states (R1, R2, R3, R4) and four within‐scanner questionnaires (AS1, AS2, AS3, AS4), carried out in two sessions separated by about 50 min. The first session started with the first questionnaire (AS1) and was followed by the first resting state (R1). Thereafter, two movement tasks (self‐paced button press and stimulus‐paced button press using a visual prompt in intervals of 10 s, each lasting 600 s) were performed. The first session ended with the second resting state (R2) and second questionnaire (AS2). Filling out each questionnaire took approximately 5 min, and each resting state lasted for about 350 s. The second session was a duplicate of the first one, with two resting states (R3, R4) and two within‐scanner questionnaires (AS3, AS4). Individuals were requested to keep their eyes open, to stay awake and to avoid movements during the resting states.

The state anxiety was assessed with the state‐trait anxiety and depression inventory (STADI; Laux *et al*., 2013), which was presented on a screen within the scanner. The STADI is an instrument constructed to assess both state and trait aspects of anxiety and depression. It is based on the State‐Trait Anxiety Inventory (Spielberger *et al*., 2009) but allows a reasonable separation of anxiety and depression symptoms. Items were answered with a trackball following each resting state.

### ECG recording and RRI time courses

ECG was recorded inside the scanner using the Siemens Physiological ECG Unit. For the positioning of the ECG electrodes on the thorax, standard channels (Siemens Standard, lead 1) were used. The respiratory data were acquired using a pneumatic cushion which is connected via an air hose to a pressure sensor on the PERU‐unit. The cushion is attached to the subject using a respiration belt. The sampling rate used was 400 Hz. The fMRI plug‐in for EEGLAB (Niazy *et al*., 2005) was used to detect ECG beat‐to‐beat complexes. Within this tool, the FASTR algorithm (for removal of gradient‐induced artifacts) and the beat‐to‐beat detection algorithm were used in succession, resulting in beat‐to‐beat (RRI) interval time courses. These were then interpolated to the same sampling frequency as the BOLD acquisitions (1/871/ms).

### fMRI and phase‐locking calculation

Functional images were acquired with a 3T scanner (Magneton Skyra; Siemens, Erlangen, Germany) using a multiband GE‐EPI sequence (Moeller *et al*., 2010) with a simultaneous six‐band acquisition with TE/TR = 34/871 ms, 52° flip angle, 2 × 2 × 2 mm³ voxel size, 66 contiguous axial slices (11 × 6), acquisition matrix of 90 × 104 and a FOV of 180 × 208 mm². For the fMRI resting state time series, 400 repetitions of the 66‐slice volumes were acquired. Preprocessing, which included slice‐timing correction adapted for multiband acquisitions (Woletz *et al*., 2014), motion correction (realignment), normalization to MNI space, resampling to 3 × 3 × 3 mm^3^ isotropic voxels (for compatibility with the spatial resolution of the atlas template provided with the toolbox), and smoothing with a 4 mm FWHM Gaussian kernel and linear detrending, was performed using the DPARSFA toolbox (Yan & Zang, 2010). To avoid the suppression of any meaningful variance around 0.1 Hz, only SPM standard realignment (rigid body transformation) was performed for all subjects. Standard procedures to correct for non‐neural BOLD signals (Fox *et al*., 2007; Power *et al*., 2012; Snyder & Raichle, 2012) were skipped because they were deemed to be potentially counterproductive in this context. Only framewise displacement (FD) (Power *et al*., 2012) was computed to rule out that any subsequent results might be confounded by motion artifacts. Finally, the AAL atlas (Tzourio‐Mazoyer *et al*., 2002) was used to extract time courses from 26 ROIs: precentral gyrus (1, 2), middle frontal gyrus (7, 8), middle frontal gyrus, orbital part (9, 10), inferior frontal gyrus, orbital part (15, 16), supplementary motor area (19, 20), superior medial frontal gyrus (23, 24), medial frontal gyrus, orbital part (25, 26), insula (29, 30), anterior cingulum (31, 32), middle or midcingulum (33, 34), posterior cingulum (35, 36), amygdala (41, 42) and precuneus (67, 68). The numbers indicate the ROI labels according to the AAL atlas. Odd and even numbers denote left and right hemispheres, respectively.

Wavelet transform coherence was applied to the BOLD and RRI time series using the ‘Cross Wavelet and Wavelet Coherence toolbox’ (Grinsted *et al*., 2004). A Morlet mother wavelet was chosen. Wavelet transform coherence provides a time–frequency map of complex coherence between two signals. We focus on the phase component, which allows us to compute the PLV throughout the acquisition interval except for small sections at the beginning and end, where results are known to be unreliable (Torrence & Compo, 1998). PLV is a normalized measure of how much the phase difference between two signals changes in a user‐chosen time window, regardless of the actual phase difference value (Lachaux *et al*., 1999). This computation was restricted to frequencies between 0.07 and 0.13 Hz and was performed for every time point with a window size of four cycles (corresponding to about 40 s). To compute the statistical significance of PLV values and thereby to test the null hypothesis of independent pairs of oscillatory activity, a surrogate‐based method was used (Hurtado *et al*., 2004). For further details, see Pfurtscheller *et al*. (2017a,2017b). Mean time delays were computed for pairs of RRI time courses and BOLD signals (left MCC) in each subject and rest epoch. Crucially, averaging was restricted to values for which PLV was significant (*P* < 0.05). The obtained such values were the basis for the distinction between negative and positive time delays (nTD and pTD, respectively). The percentages of significant (*P* < 0.05) time samples, or bins (%sigbins), which indicate the total length of a significant phase‐locking episode, were also computed in each case.

Phase‐locking value profiles restricted to the frequency band of interest (0.07–0.13 Hz) were calculated for BOLD pairs from 26 ROIs and averaged across all time points, yielding a single mean PLV value for each pair. These were then stored as 26 × 26 connectivity matrices, separately for different groups and used as input to the GraphVar package (Kruschwitz *et al*., 2015). This package applies a two‐sample *t*‐test for group comparisons, yielding 26 × 26 matrices showing significant intergroup differences. GraphVar resorts to a permutation‐based non‐parametric approach with a user‐defined number of randomly reassigned groups (we used *N* = 300) to establish a null hypothesis distribution. The *P*‐values that express the probability of a correct rejection of the null hypothesis of no intergroup difference are based on this distribution.

## Results and statistical analyses

### State anxiety

**Figure 1:**
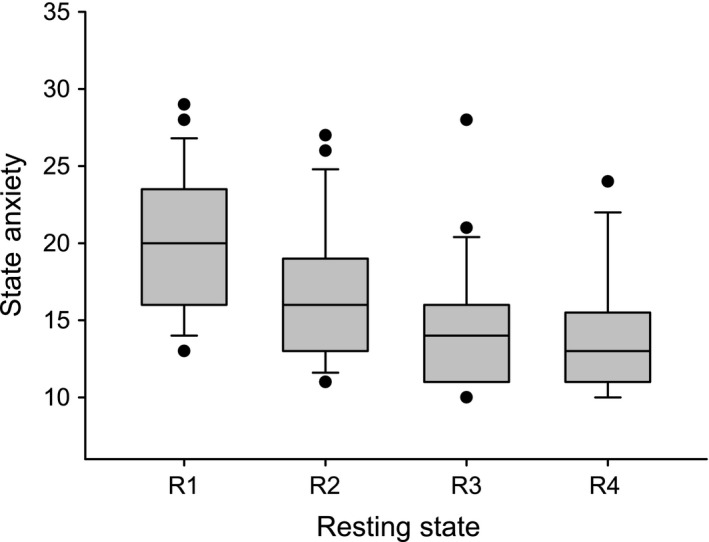
Boxplot depicting the distribution of state anxiety across the four resting states (R1–R4). State anxiety significantly declined throughout the resting states with all comparisons being significant (*P *<* *0.05), except R3–R4 (*P* = 0.16). *Note:* whiskers indicate the 10th and 90th percentile.

The state anxiety varied across participants and resting states (R1, R2, R3 and R4) in a broad range between 10 (low anxiety) and 29 (rather high anxiety) (possible range of scores: 10–40) and declined from R1 (AS1 = 20.0 ± 4.5) to R4 (AS4 = 14.0 ± 4.0) significantly [*t* (22) = 4.56, *P* = 0.001] (Fig. 1). Of note, initial state anxiety (AS1) was significantly higher than in the normative sample (Laux *et al*., 2013), namely *M* = 20.0, SD = 4.5 vs. *M*_normative sample_ = 16.3, *t* (22) = 3.91, *P* = 0.001, thus, suggesting comparably high levels of anxiety in the first resting state R1.

### Phase‐coupling between 0.1‐Hz oscillations in RRI time courses and BOLD signals

**Figure 2:**
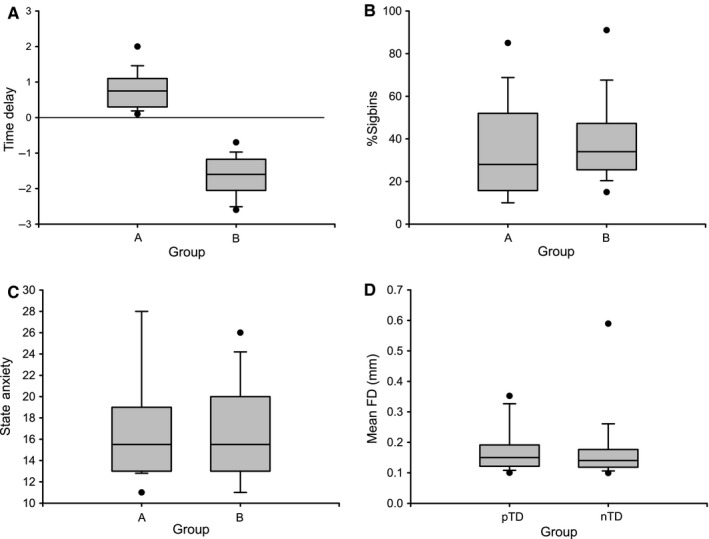
Boxplots (A, B, C and D) contrasting 18 individuals (two subgroups) in time delay (A), %sigbins (B), state anxiety (C) and framewise displacement (D).

Our recent study (Pfurtscheller *et al*., 2017a,2017b) has demonstrated that the direction of time delay (TD) between RRI oscillations and BOLD oscillations in MCC at 0.1 Hz distinguishes between neural and vascular BOLD fluctuations. Therefore, the search for neural (pTD) and vascular (nTD) BOLD oscillations was based on calculation of the PLV between RRI and BOLD signals from left MCC in 23 subjects and four resting states. This search included only subjects with reliable PLV, where reliability was defined as the length (%sigbins) of significant (*P* < 0.05) phase‐locking episodes with %sigbins ≥ 10%. Reliable nTDs were observed in 22 subjects and reliable pTDs in twenty subjects. Noteworthy, both, reliable pTD and nTD, were found in 18 subjects, meaning that one subject can show nTD in one resting state and pTD in another resting state. From the 18 subjects, two groups were formed, one with pTD (group A) and another with nTD (group B) and three features (time delay, coupling length and state anxiety) studied in detail. The corresponding boxplots (Fig. 2A, B and C) illustrate that despite the large difference between both groups in time delay (mean ± SD): pTD = 0.82 s ± 0.48; nTD = −1.71 s ± 0.61; *t* (17) = 12.99, *P* < 0.001), there was no difference in neither coupling length (%sigbins) nor state anxiety. This suggests that both high or low anxiety states can be associated with dominant neural or vascular BOLD oscillations. The comparison of the framewise displacement (FD) in both groups is illustrated in Fig. 2D and revealed no difference (pTD: M = 0.17 mm ± 0.07; nTD: M = 0.17 mm ± 0.11); *t* (17) = 0.013, *P* = 0.99.

**Figure 3:**
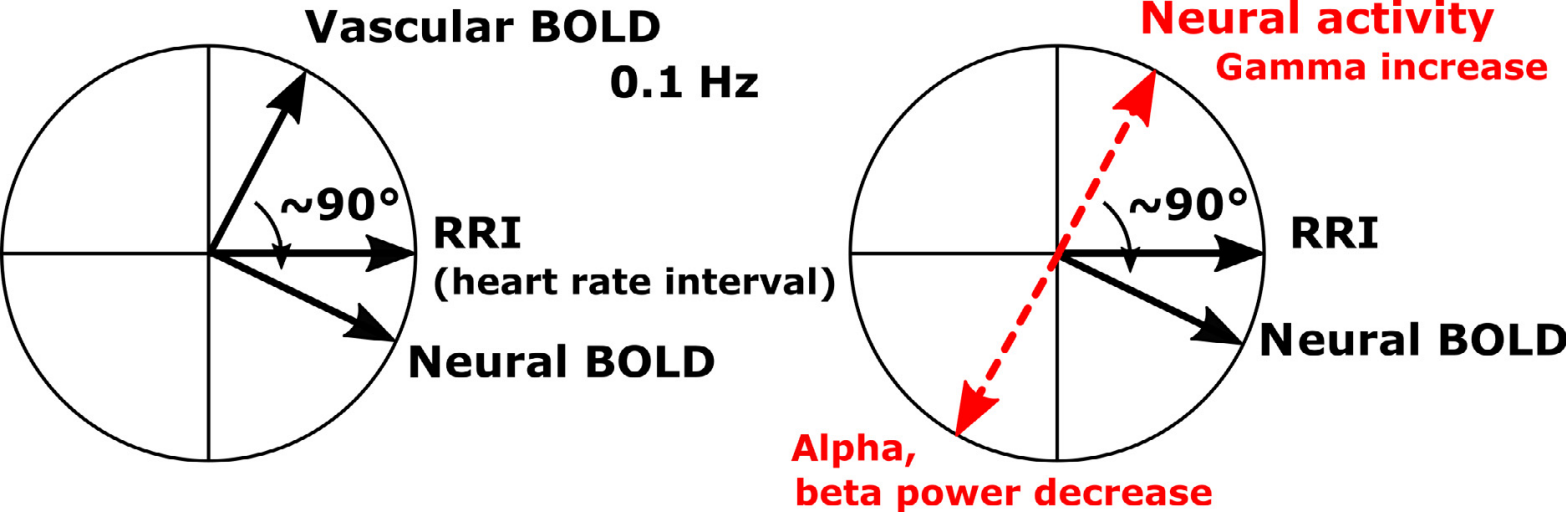
Left: Phasor model showing phase relationships between intrinsic 0.1‐Hz RRI, neural oscillations and vascular oscillations in 18 subjects. b the 90° phase shift between vascular and neural 0.1‐Hz BOLD oscillations. Right: phasor model for predicted neural and EEG power oscillations, respectively. An increase in neural activity is generally associated with a decrease in alpha and/or beta power (ERD) (Pfurtscheller & Lopes da Silva, 1999) and a gamma increase (Logothetis & Wandell, 2004). [Colour figure can be viewed at wileyonlinelibrary.com].

Phase‐coupling between oscillations can be determined by time delays (Fig. 2A) or phase shifts. In the latter case, the phasor model (Zheng *et al*., 2010; Pfurtscheller *et al*., 2017a) is best suitable to display phase shifts between slow hemodynamic (BOLD), neural and RRI oscillations (Fig. 3). One circle (360°) in the phasor model represents one 10‐s period of a 0.1‐Hz oscillation. The RRI oscillations precede the neural BOLD oscillations by 29° (corresponds to 0.8 s) and lag the vascular BOLD oscillations by 61° (corresponds to −1.7 s). The sum of the difference between both types of BOLD oscillations is 90° or 2.5 s at 0.1 Hz with vascular BOLD oscillations leading. If a neurovascular coupling time of 2.5 s is assumed (Golanov *et al*., 1994; Fox *et al*., 2007; Bruyns‐Haylett *et al*., 2013), the predicted neural activity precedes the neural BOLD oscillation by 90° (Fig. 3 right).

The relationship of the numbers of vascular (nTD) and neural (pTD) BOLD oscillations across the four resting states is summarized in Table 1. While the state anxiety declined from R1 to R4 (Fig. 1), the number of subjects with pTD and nTD indicates no clear trend. It is noteworthy that the number of subjects displaying pTD varied between 17% and 43% across resting states, while the number of subjects displaying nTD varied from 43% in R1 to 65% in R4. No significant changes were found. Many subjects revealed unreliable PLV (%sigbins =< 10%) in different resting states often caused by artefacts in the RRI time courses, due to the ECG recording within the scanner.

**Table 1:** Numbers and percentages of subjects with reliable pTD and nTD (%sigbins ≥ 10%) and with unreliable TD (%sigbins < 10%) between BOLD (left MCC) and RRI oscillations for all four resting states

23 subjects	Resting states							
	R1		R2		R3		R4	
	*n*	%	*n*	%	*n*	%	*n*	%
pTD	7	30	10	43	8	35	4	17
nTD	10	43	10	43	12	52	15	65
Unreliable PLV	6	26	3	13	3	13	4	17

### Phase‐coupling between BOLD oscillations within groups A and B

**Figure 4:**
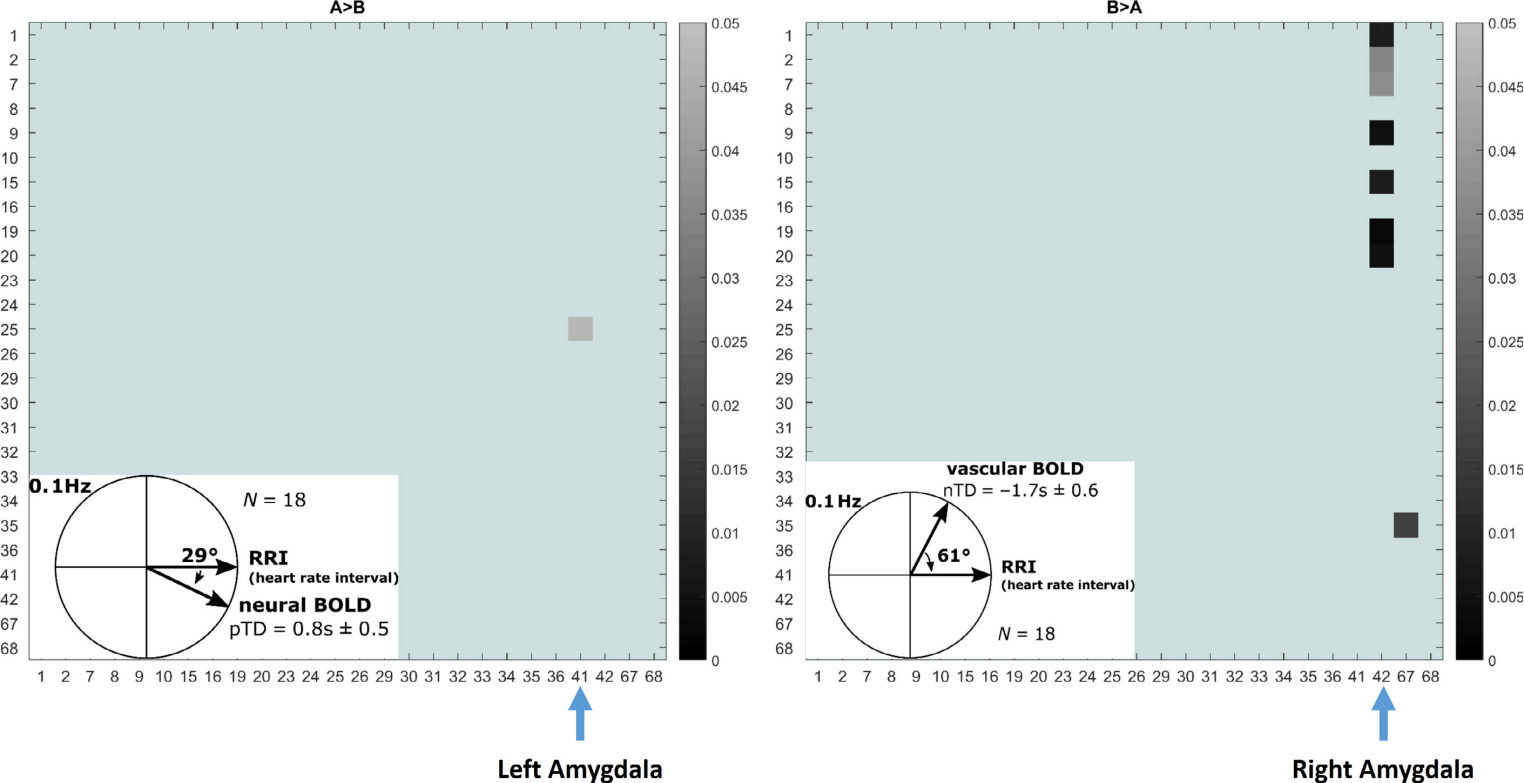
26 × 26 connectivity matrices showing significant group differences (*P* < 0.05). Left: A (pTD) > B **(**nTD) and right: B (nTD) > A (pTD). Data from 18 subjects. The corresponding phasor models for neural (left) and vascular (right) 0.1‐Hz oscillations are included. Scale for *P*‐value in gray (light gray: *P* = 0.05; dark gray: *P* = 0.01). x‐ and *y*‐axis: ROI labels according to the AAL atlas. Labels for left amygdala (41) and right amygdala (42) are indicated. [Colour figure can be viewed at wileyonlinelibrary.com].

**Figure 5:**
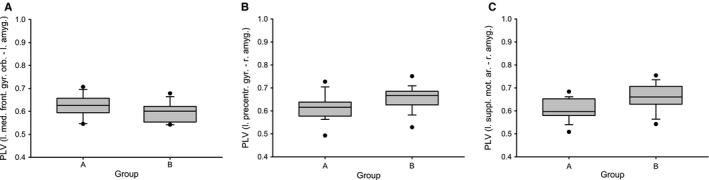
Boxplots (A, B and C) contrasting 18 individuals (two subgroups) in connectivity (PLV) between the following ROIs: left medial frontal gyrus, orbital part (25) vs. left amygdala (41) (A), left precentral gyrus (1) vs. right amygdala (42) (B) and left SMA (19) vs. right amygdala (42) (C).

To assess the validity of the heart beat‐to‐beat interval (RRI)‐based discrimination of subjects in two groups, one with dominant pTD (group A) and one with dominant nTD (group B), a connectivity analysis was performed on 0.1‐Hz BOLD oscillations with focus on amygdala and prefrontal cortical structures (Phelps *et al*., 2001; Davidson, 2002; Morawetz *et al*., 2017). To verify this discrimination, mean PLVs were calculated for BOLD pairs from 26 ROIs in prefrontal region and amygdala and the intergroup differences between neural (pTD) and vascular (nTD) BOLD oscillations tested. To highlight the relationship between both groups defined by RRI‐based discrimination (group A and group B) and BOLD connectivity analyses, the corresponding phasor models were inserted in both 26 × 26 difference matrices. The difference in 0.1‐Hz connectivity between left amygdala (41) and left medial frontal gyrus, orbital part (25) for group A (pTD) > group B (nTD) (Fig. 4 left) was significant (*P* < 0.05). In the case of B (nTD) > A (pTD), the difference (Fig. 4 right) was significant (*P* < 0.01) between right amygdala (42) and left precentral gyrus (1), left middle frontal gyrus, orbital part (9), left inferior frontal gyrus, orbital part (15) and both supplementary motor areas (19, 20). In addition, a significant coupling was found between left posterior cingulum (35) and left precuneus (67). Remarkably, both neural and vascular BOLD oscillations are accompanied by connectivity patterns with nearly exclusive involvement of the amygdala. Only one single functional connectivity was found in the case of neural BOLD oscillations, namely between left amygdala and left medial frontal gyrus, orbital part (Fig. 4 left). Multiple connectivities's were found for vascular BOLD oscillations between right amygdala, orbitofrontal cortex, supplementary motor area and lateral precentral cortices. To underline the significant group differences documented in Fig. 4, boxplots from the 18 subjects and three representative ROI pairs (25–41, 1–42 and 19–42) are displayed in Fig. 5. The corresponding Cohen's *d* revealed *d* = 0.38, 0.58 and 0.76. The effect size was medium for the pairs 25–41 (left medial frontal cortex vs. left amygdala) and 1–42 (left precentral gyrus vs. right amygdala), and great for the ROI pair 19–42 (left SMA vs. right amygdala) (Cohen, 1988).

### 0.1‐Hz BOLD oscillations in amygdala

**Figure 6:**
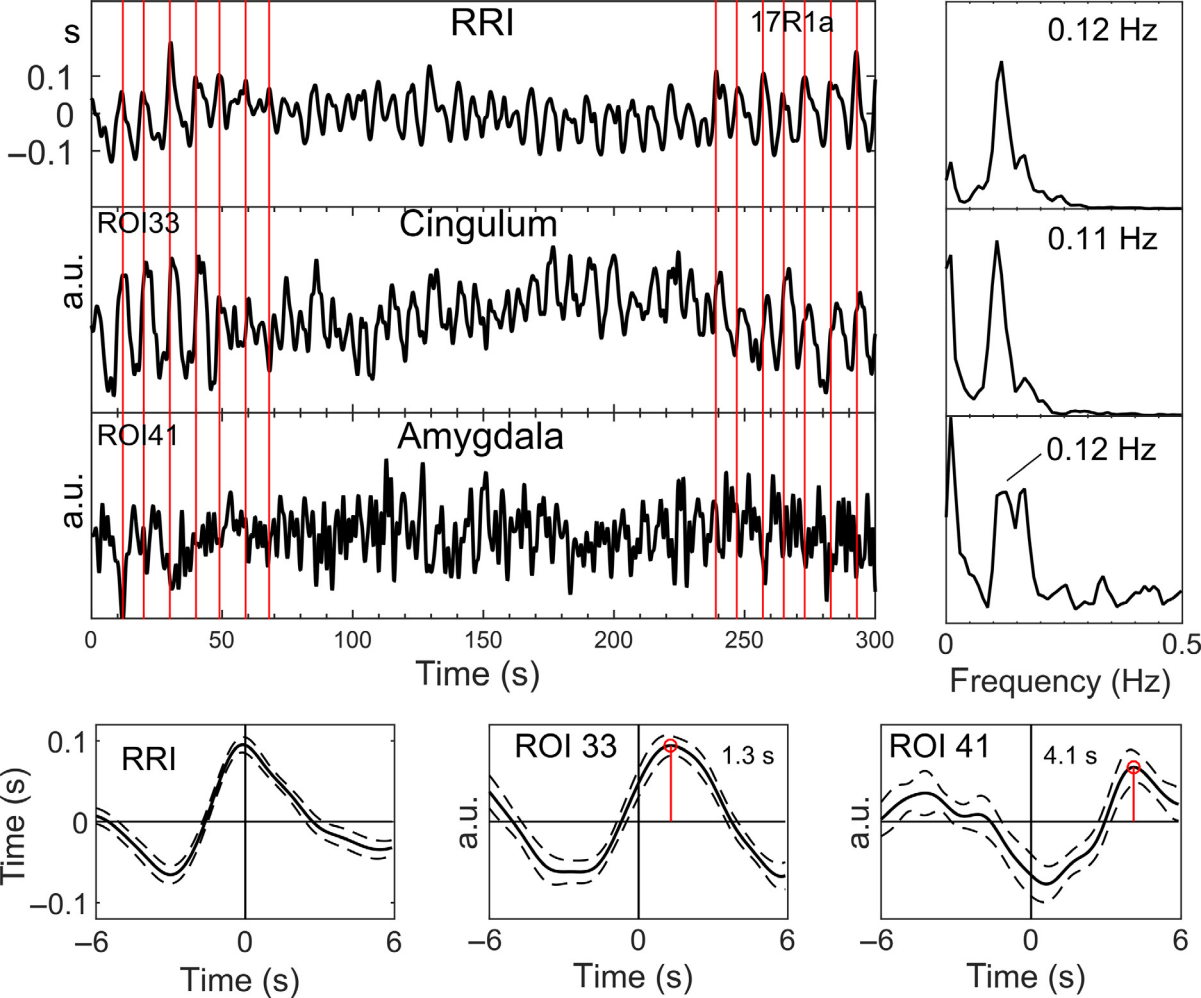
Time courses of RRI and BOLD signals from MCC and amygdala (top left), power spectra (top right) and RRI peak‐triggered averages (± SE; bottom) of RRI and BOLD signals from one subject (s17) with neural BOLD oscillations (pTD) in rest R1. The vertical lines indicate the triggers. The 0.12‐Hz oscillations in amygdala are superimposed by a signal with dominant 0.17‐Hz rhythm. [Colour figure can be viewed at wileyonlinelibrary.com].

**Figure 7:**
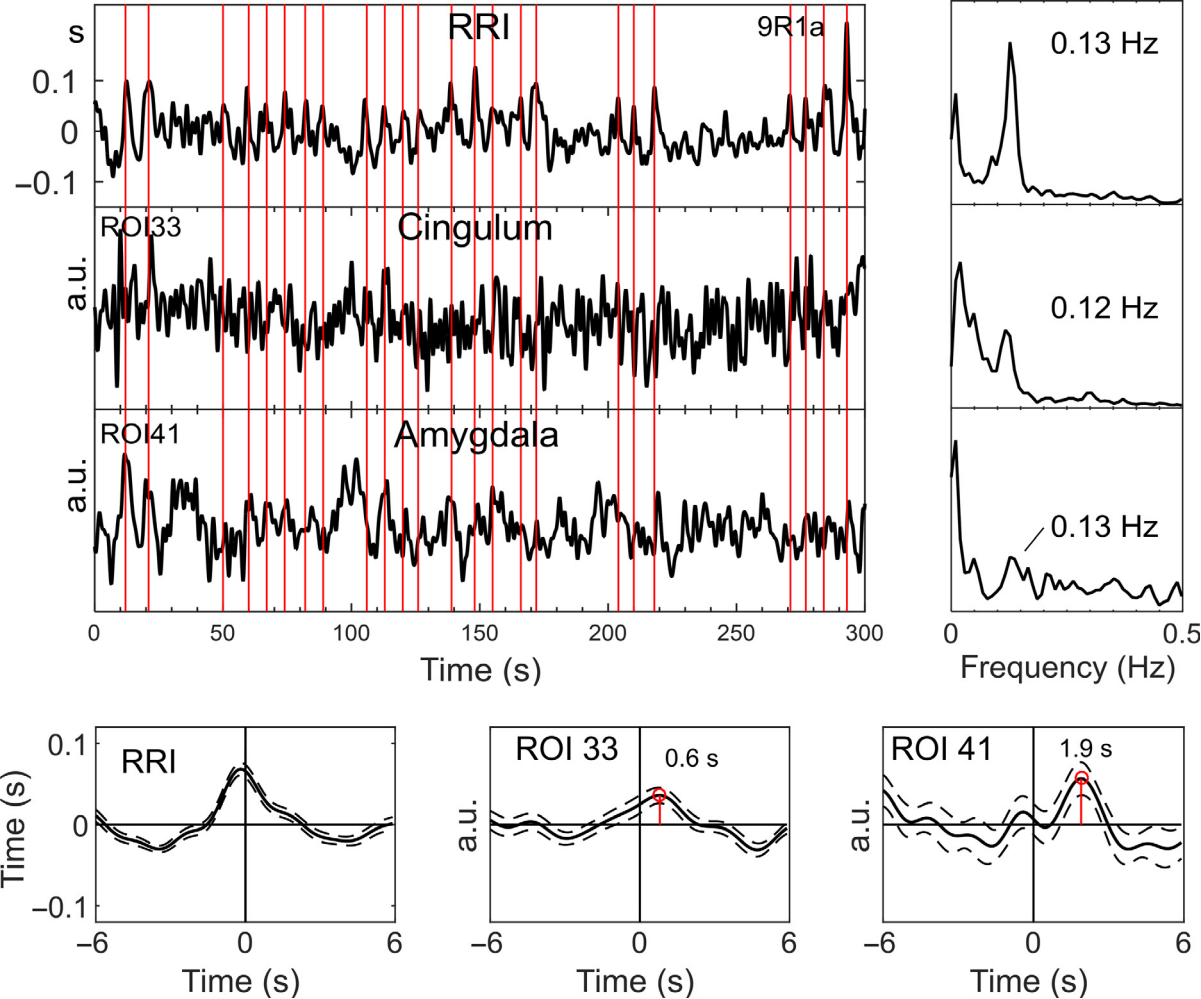
Time courses, signals and spectra from subject s9. For further explanation, see Fig. 6. The 0.13‐Hz oscillations in the amygdala are distinguishable, lagged to RRI and cingulum BOLD and are disturbed by other oscillations. [Colour figure can be viewed at wileyonlinelibrary.com].

To illustrate the existence of intrinsic neural 0.1‐Hz BOLD oscillations in amygdala and cingulum (the latter is used for selection of neural BOLD oscillations), characteristic examples from two subjects are displayed in Figs 6 and 7. Depicted are signals, time courses of RRI peak‐triggered averaged waves and power spectra. Because of the lack of triggers in resting state data, RRI peak‐triggered averaging was used (Pfurtscheller *et al*., 2012b, 2017a). To obtain triggers, we selected and marked a number of consecutive peaks in the RRI time course (indicated by vertical lines in Figs 6 and 7) in periods with significant phase‐coupling. The markers were used as triggers to compute average waves of 12‐s length (with 6 s before the trigger) for RRI and BOLD signals. In one example (Fig. 6, s17), the breathing rate was approximately 6/min and in the other example (Fig. 7, s9) about 18/min (signals are not shown). While the periods of breathing but also of RRI and BOLD waves changed between ~9 s (0.11 Hz) and ~6 s (0.17 Hz) in s17 (peaks in spectra at 0.11/0.12 Hz and 0.17 Hz), the breathing rate was relatively stable at 0.3 Hz in s9 and the RRI and BOLD signals showed oscillations dominantly at 0.12/0.13 Hz. Noteworthy is also the leading of RRI before BOLD waves in both subjects, characteristic for BOLD oscillations of neural origin. The lead of the BOLD waves in cingulum prior to those in amygdala in both cases suggests that neural activity spreads slowly from cingulum to amygdala. The BOLD signal in the amygdala is superimposed by a signal with a frequency at 0.17 Hz in s17 and is disturbed in s9, although the slow 8‐s cycle (0.13 Hz) can be identified. Remarkably, frequency components > 0.1 Hz (peaks in BOLD spectra), suggesting the existence of rhythmic central commands and neural BOLD oscillations, respectively, were not only found in two subjects s17 and s9, but in the majority of participants.

## Discussion

The study yielded two main findings, one referring to intrinsic neural BOLD oscillations at 0.1 Hz and the other to brain connectivity. Both findings are related to each other, because brain connectivity analysis depends on the direction of the time delay (pTD, nTD) between BOLD and RRI oscillations. In the following paragraphs, these two main findings will be discussed in separate sections.

### Subjects with neural 0.1‐Hz BOLD oscillations (pTD) lagging those in RRI signals

Neural BOLD oscillations were found in about 80% of subjects at least in one of the four resting states. This is in strong contrast to the 17 to 43% of subjects with neural BOLD oscillations found in each resting state (R1 to R4; Table 1). This difference is due to the observations that in each resting state, the number of subjects with neural and vascular BOLD oscillations was alternating, and neural oscillations were most frequent in the second resting state R2 (10 subjects; see Table 1) and least frequent in the last resting state R4 (4 subjects). This means that nTD and pTD can vary across resting states in individual subjects, a finding described as ‘switching phenomenon’ (Pfurtscheller *et al*., 2017a). For example, pTD in one subject in R1 switched to nTD in R2 and nTD in another subject in R1 switched to pTD in R2. The observed switching together with the finding of a declining state anxiety from R1 to R4 suggest that during conscious rest, two strategies may exist to manage the processing of anxiety: (i) an increase in slow rhythmic neural activity (neural BOLD, pTD) and (ii) an increase in blood circulation (vascular BOLD, nTD). In this context, two issues deserve consideration. First, in contrast to the work of Julien (2006), who challenged the existence of a central pacemaker for the Mayer waves in blood pressure, our study provides support that central commands (neural BOLD oscillations), which occurred in the majority of subjects, modulate the heart rate rhythmically at intervals of about 10 s. Second, neural BOLD oscillations are present at frequencies below and above 0.1 Hz (see examples in Figs 6 and 7), which contradicts the popular opinion that oscillations with frequencies above 0.1 Hz are artifacts (Snyder & Raichle, 2012).

### Phase shift between neural and vascular BOLD oscillations at 0.1 Hz in midcingulum

Two theories have been proposed to explain and discuss the frequency of Mayer waves (0.1 Hz) in arterial blood pressure and heart rate: the baroreflex theory and the pacemaker theory (for details see Julien, 2006). While the former is related to the baroreflex loop, the latter is based on central commands originating in prefrontal cortex and modulating the heart rate via fast vagal nerve activation (Thayer & Lane, 2009). The influence of Mayer waves in heart rate and RRI, respectively, determines the weight of the low‐frequency HRV (0.04–0.15 Hz). In general, a high HRV signifies high autonomic flexibility, a type of resource important for emotional processing (Appelhans & Luecken, 2008; Lagos *et al*., 2008; Thayer & Lane, 2009). Although the origin of low‐frequency HRV in blood pressure and HR is still mostly unknown (Kuusela *et al*., 2003), a high HRV can be expected when both components (related to the baroreflex loop and cortical control of cardiac activity by central commands) appear at the same moment of time. This means that slow oscillations in the circulation (vascular BOLD) and slow oscillations in prefrontal neural activity associated with hemodynamic changes (neural BOLD) should be approximately time‐locked. If we accept this assumption, the observed phase shift of ~90° (or ~2.5 s) for 0.1 Hz oscillations between vascular and neural BOLD oscillations in the group of 18 subjects (Fig. 3) can be interpreted as an estimate for the neurovascular coupling time during rest. The question about a possible link between natural slow rhythmic (~0.1 Hz) neural and BOLD changes was discussed by Mateo *et al*. (2017). They reported that oscillations in arteriole diameter, entrained by intrinsic gamma power fluctuations, coherently drive fluctuations in blood oxygenation. Interestingly, the reported time delay between gamma power increase and BOLD increase is almost identical (actually ~2.6 s) to our delay of ~2.5 s between vascular and neural BOLD oscillations.

Vascular BOLD oscillations at 0.1 Hz are associated with cerebral blood circulation. Therefore, it is not unexpected that spatial patterns of slow frequency oscillations between 0.01 and 0.2 Hz in a resting state study with ultrafast fMRI (TR = 0.4 s) were explained as result of cerebral blood circulation (Tong & Frederick, 2014). Such cerebral blood flow oscillations with 4–8 waves/min (Mayer waves) are characteristic for the cerebral blood flow velocity (CBFv) measured with transcranial Doppler sonography in middle cerebral artery and are phase‐coupled with finger arterial blood pressure oscillations (Diehl *et al*., 1998; Zhang *et al*., 1998). These slow CBFv oscillations precede not only the blood pressure oscillations but also the RRI oscillations (De Boer *et al*., 1998; Kuusela *et al*., 2003; Pfurtscheller *et al*., 2011) and can be seen as a driving force of vascular BOLD oscillations.

### Phase‐coupling between BOLD oscillations at 0.1 Hz

One purpose of the comparison of phase‐coupling matrices with dominant neural (group A) vs. vascular (group B) 0.1‐Hz BOLD oscillations was to examine whether different patterns are found for amygdala and prefrontal activation in anxious subjects. The result of this comparison revealed significant intergroup differences for comparisons A > B as well as B > A (Figs 4 and 5) and corroborate that the distinction between neural and vascular BOLD oscillations, based on phase‐coupling between BOLD and RRI oscillations, is feasible. In the case of the comparison A (pTD) > B (nTD), the neural BOLD oscillations (pTD) revealed enhanced connectivity between left amygdala and left medial frontal cortex (Fig. 4 left), while the comparison B (nTD) > A (pTD) showed enhanced connectivity of vascular BOLD oscillations (nTD) between right amygdala and multiple regions in left orbitofrontal cortex, left supplementary area and left precentral and middle frontal gyri (Fig. 4 right). The enhanced connectivity between amygdala and medial prefrontal cortex is in agreement with the findings of Urry *et al*. (2009), Veer *et al*. (2011), Vytal *et al*. (2014) and others, who have shown that the amygdala and the medial prefrontal cortex are activated during processing and regulation of negative emotions. The synchronization of slow neural BOLD oscillations at 0.1 Hz in the amygdala and the medial prefrontal cortex is a novel finding.

In contrast to neural BOLD oscillations, which showed only one significant pair of coupling, vascular BOLD oscillations revealed multiple couplings, all of them involving the right amygdala. These highly significant couplings included regions in the supply territory of the anterior cerebral artery (supplementary motor area), the middle cerebral artery (precentral gyrus, left middle frontal gyrus) and the anterior choroidal artery (amygdala). The coupling of right amygdala with left prefrontal and left middle frontal areas is particularly astounding, however, confirmed by the higher perfusion in the supply territory of the left middle cerebral artery during high state anxiety (Pfurtscheller *et al*., 2017b). Whether the circle of Willis connecting the blood supply of both hemispheres or other factors plays a role need further research. The multiple coupling pattern with vascular BOLD oscillations suggests that brain regions involved in the processing of fear and/or anxiety require increased cerebral blood flow to match the required increased metabolism in these regions. The mechanism responsible for adequate blood supply is cerebral autoregulation (Zhang *et al*., 1998), which adjusts the cerebral arteriolar caliber of the different branches of cerebral arteries involved in emotional processing. Vascular BOLD oscillations could therefore be used to track bulk blood flow in the brain as suggested by Tong & Frederick (2012).

### Limitations and future prospects

As the Nyquist frequency for the BOLD signal is around 0.5 Hz, there could still be aliasing due to the heart signal introducing distortion in the observed frequency band. Head motion definitely is a problem if the connectivity of BOLD signals is studied in the frequency band 0.009 Hz–0.08 Hz, because BOLD signals are sensitive to subject motion and need therefore methods to reduce motion‐related artefacts (Power *et al*., 2012). In the case of studying BOLD signals in the frequency band 0.07–0.13 Hz and the comparison between subgroups, there is no reason to suppose that head motion effects would express themselves differently in these subgroups. Because of the clearly higher amount of vascular BOLD oscillations (cerebral blood circulation) in the left hemisphere (Pfurtscheller *et al*., 2017b), it could be argued that the use of for example RETROICOR (Glover *et al*., 2000) to remove artefacts might be counterproductive because it could remove the HR variability effects that we rely upon for some of our conclusions.

With respect to breathing patterns, it should be acknowledged that two types are possible: either breathing at self‐paced intervals controlled by cerebral cortex or descending autonomic (metabolic) breathing controlled by centers in the brain stem. The latter is not only controlled by metabolic commands but also by changes in emotion, such as for example anxiety (Homma & Masaoka, 2008). The finding of 0.17‐Hz waves in BOLD signals from amygdala, RRI time courses (Fig. 6) and respiration (not shown) is a first hint for a relationship between autonomic breathing and anxiety processing. Confirmation about the possible source of 0.17‐Hz oscillations in the brain stem came from the work of Perlitz *et al*. (2004). They introduced the term ‘0.15‐Hz rhythm’ to describe cardiovascular‐respiratory oscillations in the band 0.12–0.18 Hz in the brain stem, phase synchronized with respiration. We found such a rhythm predominately at 0.17 Hz, alternating with other breathing rates in few individuals. Due to the electrical source of this ‘0.15‐Hz rhythm’ in the reticular formation (Perlitz *et al*., 2004), it is very likely that BOLD oscillations in regions close to the brain stem, as for example amygdala, absorb this rhythm (see example in Fig. 6). Further experiments should focus on this relationship and the source of 0.17‐Hz oscillations in BOLD signals.

The amygdala is an important part of the limbic system and crucial to maintain anxious states (Vytal *et al*., 2014). BOLD signals from the amygdala are often confounded by magnetic inhomogeneity and a low signal‐to‐noise ratio. They can be disturbed by nearby blood vessels (Birn *et al*., 2008) and are also less smooth by the low number of voxels when the AAL atlas is used (Korhonen *et al*., 2017). Examples of two individuals, one with 6/min respiration (Fig. 6) and one with 18/min respiration (Fig. 7), show in the first case frequency components at 0.11/0.12 Hz and 0.17 Hz in the BOLD spectrum and in the second case frequency components at 0.12/0.13 Hz with a relatively disturbed BOLD signal in the amygdala. Nevertheless, despite of the dissatisfied quality of raw BOLD signals from amygdala, there was evidence for a significant rhythmic phase‐coupling in the group of spontaneous neural BOLD oscillations at 0.1 Hz between medial frontal cortex and amygdala.

It has to be kept in mind that no instruction was given to the participants about the type of respiration. Nevertheless, a minority of individuals displayed a spontaneous breathing at 6/min only in the first resting state where the state anxiety was highest. This may be not astonishing when it is taken into consideration that breathing at 6/min (0.1 Hz) approximates the resonance frequency of the human baroreflex loop (Lehrer, 2013) and results in a very large HRV favorable to manage the processing of unpleasant emotions (Homma & Masaoka, 2008; Thayer & Lane, 2009). Breathing at 6/min could be seen therefore as one strategy to regulate high anxiety levels.

## Conclusion

1. Neural BOLD oscillations in the 0.1‐Hz frequency range were found in the majority of subjects (about 80%). They comprise a neural component that most likely is directly correlated with electrophysiological oscillations in the slow/infra‐slow frequency band (Foster & Parvizi, 2012; Palva & Palva, 2012). They reflect fluctuations in cortical excitability, which are considered as a basic characteristic of brain activation during rest (Vanhatalo *et al*., 2004).
2. Neural BOLD oscillations can be dissociated from vascular BOLD oscillations on the basis of systematic phase shift differences relative to heart rate (RRI) oscillations. The two BOLD components exhibit a phase shift of approximately 90° (or 2.5 s) at 0.1 Hz, with vascular oscillations leading. This time lag corresponds to the neurovascular coupling time during rest (Golanov *et al*., 1994; Fox *et al*., 2007; Bruyns‐Haylett *et al*., 2013; Mateo *et al*., 2017). The time‐locked behavior of vascular BOLD oscillations (Mayer waves) and slow neural oscillations preceding those in neural BOLD oscillation by about 2.5 s contribute to an enhanced HRV and represent an important resource for emotional regulation (Thayer & Lane, 2009).
3. Our results also suggest that 0.1‐Hz neural BOLD oscillations can be dissociated from vascular BOLD oscillations by connectivity measures (e.g. phase‐coupling) between BOLD oscillations in amygdala and related regions known to be important for the processing of unpleasant emotions. This dissociation suggests that two strategies exist to optimize the processing of anxiety: first, functional coupling between amygdala and medial frontal cortex through enhanced 0.1‐Hz oscillations (neural BOLD) and second, increased cerebral blood circulation (vascular BOLD) in amygdala and related structures.

## Conflict of interest

The authors declare no competing financial interest.

## Author contributions

GP designed research; GP, AS and AA performed research; CB, AA, ASP, JC, JG and CA analyzed data; GP, AS, AA and CB wrote the paper.

## Data accessibility

Data used in this article will be made available upon request to the corresponding author.

## Abbreviations

AAL: automated anatomical labeling
BOLD: blood‐oxygen‐level‐dependent
CBFv: cerebral blood flow velocity
ECG: electrocardiogram
EEG: electroencephalogram
ERD: event‐related desynchronization
fMRI: functional magnetic resonance imaging
HRV: heart rate variability
MCC: midcingulum
nTD: negative time delay
PLV: phase‐locking value
pTD: positive time delay
ROI: region of interest
RRI: heart beat‐to‐beat interval
STADI: state‐trait anxiety and depression inventory
