# Supplementary material for: Synchronization of intrinsic 0.1‐Hz blood‐oxygen‐level‐dependent oscillations in amygdala and prefrontal cortex in subjects with increased state anxiety
Source: Eur J Neurosci. 2018 Feb 8;47(5):417–26. doi: 10.1111/ejn.13845 (PMC5887876; doi:10.1111/ejn.13845)
Supplement: Supplementary file 1 [file EJN-47-417-s001.pdf]

EJN European Journal of Neuroscience FENS Federation of European Neuroscience Societies

Synchronization of intrinsic 0.1-Hz blood-oxygen-level dependent (BOLD) oscillations in amygdala and prefrontal cortex in subjects with increased state anxiety

Gert Pfurtscheller, Andreas Schwerdtfeger, Annemarie Seither-Preisler, Clemens Brunner, Christoph Stefan Aigner, João Calisto, João Gens & Alexandre Andrade

Review timeline:

Submission date:	12 May 2017
Editorial Decision:	30 May 2017
Revision received:	07 July 2017
Editorial Decision:	31 October 2017
Revision received:	15 November 2017
Editorial Decision:	05 December 2017
Revision received:	16 January 2018
Accepted:	18 January 2018

Editor: John Foxe

1st Editorial Decision 30 May 2017

Dear Dr. Brunner,

Thank you for submitting your work to the European Journal of Neuroscience. Unfortunately, the quality of the illustrations in your paper is insufficient for a full and reasonable evaluation of the work at this stage: we request more detailed and better resolution figures to proceed to a full formal review.

We recommend that you read our recent editorial on graphical representations and reporting of results, which you can find here:

Rousselet, G.A., Foxe, J.J. & Bolam, J.P. (2016) A few simple steps to improve the description of group results in neuroscience. The European journal of neuroscience, 44, 2647-2651.

http://onlinelibrary.wiley.com/doi/10.1111/ejn.13400/full

While we are declining the paper at this stage, we would be happy to consider a version that is responsive to the issues raised above. Of course, such a version would then need to be reviewed fully by a set of independent external reviewers. We do hope you will take up the challenge of better illustrating what we believe could prove to be a very interesting study.

Here are more detailed comments on your submission:

Most distributions leading to key conclusions, and whether significant or not, must be illustrated using scatterplots.

The description of effects as significant or non-significant is unsatisfactory. Some measures of effect sizes and uncertainty should be reported in addition to p values.

A star system to indicate categories of p values is unacceptable. If you wish, use one star to indicate all p values crossing an arbitrary threshold.

What are the values reported with +/-, for instance in Table 1? For anxiety, the SEM would be

inappropriate because the scores are bounded, and SEM assumes an unbounded continuous variable.

The main results presented in Figure 4 cannot be assessed because they do not show individual results, or any measure of uncertainty.

Please remove the significance statement, and add the authors' contribution, a statement about conflicts of interest, and a statement about data and code sharing. See this recent EJN editorial on data sharing:

[[http://onlinelibrary.wiley.com/doi/10.1111/ejn.13234/full]]

Given that your analysis pipeline contains several arbitrary choices, it is essential that code and data are published along with your article - see the dangers of reporting only one end point of a series of forking paths:

Steegen, S., Tuerlinckx, F., Gelman, A. & Vanpaemel, W. (2016) Increasing Transparency Through a Multiverse Analysis. Perspect Psychol Sci, 11, 702-712.

Forstmeier, W., Wagenmakers, E.J. & Parker, T.H. (2016) Detecting and avoiding likely false-positive findings - a practical guide. Biol Rev Camb Philos Soc.

Thank you for submitting your work to EJN.

Kind regards,

John Foxe & Paul Bolam
co-Editors in Chief, EJN

Authors' Response 07 July 2017

Thank you for the opportunity to resubmit our manuscript. Please find the responses to the points raised by the editors below.

> Most distributions leading to key conclusions, and whether significant or not, must be illustrated using scatterplots.

We inserted a number of scatterplots and bar graphs for two Figures to demonstrate significant group differences.

> The description of effects as significant or non-significant is unsatisfactory. Some measures of effect sizes and uncertainty should be reported in addition to p values.

We now provide Cohen's d as a measure for effect size. In addition, two completely different approaches (one comparing phases of heart beat-to-beat intervals and BOLD signals, the other connectivity measures between BOLD signals) revealed significant group differences. These are strong arguments for significant group differences.

> A star system to indicate categories of p values is unacceptable. If you wish, use one star to indicate all p values crossing an arbitrary threshold.

Stars indicating categories of p values were eliminated in Table 1.

> What are the values reported with +/-, for instance in Table 1? For anxiety, the SEM would be inappropriate because the scores are bounded, and SEM assumes an unbounded continuous variable.

Anxiety scores (mean+-SD) were removed in Table 1.

> The main results presented in Figure 4 cannot be assessed because they do not show individual results, or any measure of uncertainty.

Scatterplots and bar graphs were made and summarized in an additional Fig. 5.

> Please remove the significance statement, and add the authors' contribution, a statement about conflicts of interest, and a statement about data and code sharing.

We have removed the significance statement and added the authors' contributions and a statement about conflicts of interest.
Data processing was strongly based on the WTC package (http://www.glaciology.net/wavelet-coherence) with minor adaptations and on the GraphVar package (http://rfmri.org/GraphVar) to calculate group differences of BOLD matrices. More specific information will be provided if necessary. A table with subject code, age and anxiety state in resting states R1, R2, R3 and R4 is included and could be added to the manuscript if desired.
BOLD and RRI data as well as analysis scripts used in our analyses will be made available. We have added this statement to our manuscript.

2nd Editorial Decision 31 October 2017

Dear Dr. Brunner,

Your resubmitted manuscript was reviewed by two external reviewers and the editors. The reviews collectively indicate that your work has generated new and important information. However, there are still several substantial issues that need to be clarified/resolved before we can consider your manuscript further for publication in EJN. For example, how have you accounted for the potential confound of contributions from motion artifacts. Also, please make clear how you have dealt with the multiple comparisons issue, and fully describe your statistical approach, which remains rather opaque. There are suggestions for ways to improve the informativeness of your illustrations. Please make sure to add a forthright limitations section to the paper.

Also, your data statement needs to be more detailed (i.e. needs to say where the data can be accessed) and your bar charts should be replaced with scatterplots or some other more informative hybrid depictions - see http://onlinelibrary.wiley.com/doi/10.1111/ejn.13400/epdf

If you are able to respond fully to the points raised, we would be pleased to receive a revision of your paper within 12 weeks.

Thank you for submitting your work to EJN.

Kind regards,

John Foxe & Paul Bolam
co-Editors in Chief, EJN

Reviews:

Reviewer: 1 (Enrico Glerean, Aalto University, Finland)

Comments to the Author
In this manuscript, the authors are focusing on an oscillatory component of the BOLD signal at 0.1 Hz. Specifically, they use a method that can separate vascular and neuronal BOLD oscillations using phase synchronization in a specific frequency band. The population considered are scanner naïve subjects with

EJN European Journal of Neuroscience FENS Federation of European Neuroscience Societies

Generalized Anxiety Disorder. This is an intriguing paper as there is growing evidence of oscillatory components of BOLD signal. There are however few omissions that require the authors to revise the paper to further strengthen the significance of their claims. Few readers might think that what we see here could be explained by considering other sources of artefacts that the authors ignored, such as respiration and head motion.
Specifically:
- Respiration and heart beat are linked by the vagal tone.
- Respiration affects head motion and magnetic field homogeneity, causing systematic artefacts in BOLD signal.
- Vagal tone activity is anomalous in subjects with GAD.

1. Head motion

It has been established that head motion is one of the major sources of artefacts in all MRI brain studies, especially with fMRI and functional connectivity (see Power et al 2012). The authors are not mentioning if and how motion correction was performed during preprocessing. The following issues should be resolved:

1.1 The authors should report if motion correction was performed
1.2 The authors should report the amount of framewise displacement (FD) as percentage of volumes with FD above 0.2 mm for each subject/session (see http://preprocessed-connectomes-project.org/quality-assessment-protocol/).
1.3 The authors should report if they used techniques to mitigate head motion artefacts such as one of these three: i) regression of head motion parameters, ii) ICA-AROMA, iii) FSL FIX, or other similar procedures.
1.4 If no technique for reducing the impact of motion artefact was in place, the authors should re-run preprocessing, possibly using ICA-AROMA as it seems to be the best performer http://www.biorxiv.org/content/early/2017/06/27/156380
1.5 Other techniques can be used to mitigate the effect of head motion such as scrubbing (censoring time points with FD > 0.2mm) or removal of global signal. Was any scrubbing in place? The authors should also explicitly report if global signal was removed or not.
1.6 Related to the previous point: the authors could clarify how many time points were used in estimating the PLV, given also the fact that with wavelet transform, the cone of influence greatly limits the total amount of time-points that are free from artefact (as noted also by the authors in the manuscript). The authors only mentioned 40s per time point, but they could specify the total number of time points and the amount of time point removed at the beginning and at the end due to the COI of the wavelet transform.

2 Computing average PLV
The authors mention that “averaging was restricted to values for which PLV was significant”, which can leave the reader wonder whether this is a source of bias, what was happening at the time points when PLV was not significant, and why they were discarded a priori. The author should first test if the instantaneous p-value of PLV correlates with instantaneous FD, i.e. to make sure that p-values (or better PLV effect sizes) are not co-varying with head motion. Furthermore, I would suggest to use all PLV values and rather than averaging the strongest ones, the whole distribution of PLV values can be used as fingerprint of each specific scanning session. Then multivariate test comparing distributions could then separate subjects into two groups as mentioned later in the manuscript. However, this is not necessary for this manuscript, although the authors should acknowledge in the discussion that there is a potential source of bias in considering only strongest PLV values.

3 When considering $p<0.05$ in the selection of PLV time points, the authors are not controlling for the multiple comparisons. I.e. the alpha should be 0.05/T with T the total number of time points considered. However, as this Bonferroni approach does not take into account the autocorrelation of the signal, the authors could estimate the intrinsic degrees of freedom of PLV time series and correct for those i.e. setting the alpha at 0.05/DF (if the PLV signal was white Gaussian then DF ~ T; from experience, I expect DF ~ T/10 or smaller).

4 Results in Figure 2

It is an expected result to obtain a p-value of E-10 when comparing the time delays of the two groups, as the two groups were indeed formed based on their time delays. I personally wouldn't stress the "extreme large effect size". This might also be a consequence of using only the strongest (PLV p-value < 0.05) when computing the averages. The result would have been more interesting with average PLV across all time points. However, it is not clear if these pTD and nTD values used for testing are indeed the average PLV over the p < 0.05 time points. Could the author please clarify how pTD and nTD were calculated? Furthermore, if they are in seconds, the unit measure should be added to the y axis in Figure 2.

5 Figure 2: it is redundant to have both A, B, C and D, E, F charts as they convey the same information. Mean and quartiles can be added to the panel A, B, C (e.g. by overlaying a box plot or a violin plot) and the barplots can be removed.

6 Figure 2: as the same individual is in both group A and group B, the dots from the same individual should be connected with a line to show a possible interaction. This is necessary when plotting paired data, see Figure 2 in Tracey et al 2015
http://journals.plos.org/plosbiology/article?id=10.1371/journal.pbio.1002128

7 Figure 2: a similar one-sample t-test could be done also using the mean framewise displacement of each session with pTD vs nTD to show that average motion did not correlate with pTD and nTD.

8 Results of Figure 4: there is no mention of multiple comparisons correction. Specifically, since there are 26 ROIs, the total number of comparisons are 26*(26-1)/2=325. False discovery rate correction could be used or, even better, the Network Based Statistics method for controlling FDR when comparing link weights (https://sites.google.com/site/bctnet/comparison/nbs)

9 The reader is left wondering if a standard method for computing functional connectivity between the 26 ROIs would have provided similar results. Since most of connectivity literature is based on Pearson's correlation of 0.01-0.1 Hz BOLD time series, the authors should compute a figure similar to Figure 4 using standard connectivity procedures between the same groups: group A and group B. This is interesting independently on the direction of the result: if the PLV 0.1 Hz result overlaps with standard functional connectivity techniques, the authors can further comment on the neuronal contribution of 0.1 Hz oscillations. On the other hand, if the two methods give different results, it could be that PLV is showing something more (e.g. related to Alpha and Beta ERD as seen in Figure 3 since low frequency BOLD contribution is mainly reflected at the Gamma band, cf. Logothetis' work).

10 Figure 5: same considerations as in, Figure 2 above (remove bar plots, overlay a box plot or violin plot if necessary or just the average, connect paired data points with a line)

11 The manuscript is lacking a "limitations" paragraph in the discussion section. The authors could mention at least these four limitations:
i) the authors do not discuss breathing as a possible driving factor in the neural/vascular BOLD separation. For example, the unexplained 0.17 Hz component in Figure 6 could correspond to a breathing rate of 10 breath/min which would not be too unusual during rest. Furthermore, breathing and chest movements during breathing introduce systematic head motion as well as inhomogeneity of the scanner magnetic field. Finally breathing rate is related to heart rate through the vagal tone, hence we cannot exclude that what we see here is related to heart-breathing synchronization reflected in the brain. Please refer to these two links for many references on these topics: 1] https://practicalfmri.blogspot.fi/2017/08/fluctuations-and-biases-in-fmri-data.html 2] https://practicalfmri.blogspot.fi/2014/12/concomitant-physiological-changes-as.html
ii) The size of each ROI is different with amygdala having lower amount of voxels than cingulum, hence the "less smooth" time series in Fig 6. (At 2mm istropic, AAL 33 "Cingulum_Mid_L" has 1943 voxels, while AAL 41 "Amygdala_L" has 234). In general, ROI size constitutes a source of artefact as the bigger the ROI the less consistent the ROI signal is (Korhonen 2016, http://www.mitpressjournals.org/doi/pdf/10.1162/NETN_a_00013).
iii) As the population was not normative, it should be stressed that these results might not extend to the healthy population.
iv) As the Nyquist frequency for the BOLD is around 0.5 Hz, there could still be aliasing due to the heart signal introducing distortion in the observed frequency band. The reader is left wondering what would have been the results if methods such as RETROICOR were used.

Minor comments

- The authors did not mention the number of channels in the coil used, I assume 32 since the MB sequence used is somewhat similar to that used by the Human Connectome Project.
- The authors did not mention the type of head-restraint use (I presume foam padding)
- The authors did not specify what type of methods was use to compensate for scanner drift (I presume linear or spline detrending)
- I have spotted one typo (Golanow should be spelled Golanov)

Reviewer: 2 (Bin Zhang, Shanghai Mental Health Center, China)

Comments to the Author
The current study showed that two novelty findings: Neural BOLD oscillations can be dissociated from vascular BOLD oscillations on the basis of systematic phase shift differences relative to heart rate oscillations, and 0.1-Hz neural BOLD oscillations can be dissociated from vascular BOLD oscillations by connectivity measures between BOLD oscillations in amygdala and PFC. The quality of the manuscript totally is good, and in my opinion, it could be accepted by EJN. However, several points should be clear clarified.

1. In the whole manuscript, I could not find the description about statistics, what kind of statistic methods, which software.
2.Page 4 and Page 5 about preprocessing of fMRI data, why no relignment step? why used 3 x 3 x 3 mm to normalization?
3. Table 1 should be modified.

Authors' Response 15 November 2017

Thank you very much for your kind action letter of October 31. My co-authors and I are pleased to learn that our manuscript entitled “Synchronization of intrinsic 0.1-Hz blood-oxygen-level dependent (BOLD) oscillations in amygdala and prefrontal cortex in subjects with increased state anxiety” was generally well received and we are invited to resubmit a revised version of the manuscript. Enclosed please find attached the revised submission.

First of all, we would like to thank you and both reviewers for the very constructive and helpful comments on our manuscript. In the following, we address each of the primary concerns raised by you and the reviewers in more detail; we quote the suggestions made and respond to each concern. When appropriate, we insert text passages from our revised manuscript.

We hope that all points have been dealt with in a satisfactory manner. On behalf of the co-authors, I would like to express our gratitude for your constructive and conscientious review of our work. We believe that the manuscript has gained much from the thoughtful comments and offers new insights into the neural underpinning of the 0.1 Hz BOLD oscillations. We are looking forward to hearing from you again.

Reviewer 1

We like to thank Reviewer 1 for critically reading our manuscript and the many constructive comments that helped to strengthen our assumption of the existence of spontaneous neural activity and neural BOLD oscillations at 0.1 Hz, respectively.

First of all, we noticed that we were a bit too sketchy in the description of our sample and apologize for that. In fact, we did not assess a clinical sample (GAD patients), but studied scanner-naïve healthy (normal) subjects. Hence, we expected relatively high state anxiety at their first ever scanning experience without implying clinical levels of anxiety.

The goal of our paper was to investigate a specific group of slow BOLD oscillations of neural origin (central rhythm, pacemaker-like activity) (Julian 2006) in the low frequency band at 0.1 Hz and a group of BOLD oscillations of non-neural origin related to Mayer waves (Mayer 1876) in the cardiovascular system by using two different approaches (phase-coupling between BOLD and heart rate interval oscillations and phase-

coupling between BOLD oscillations in prefrontal cortex). The investigation of neural vs. non-neural BOLD oscillations in the low frequency band at 0.1 Hz is a novel approach and therefore the standard procedures to correct for non-neural BOLD signals (Fox and Raichle, 2007; Snyder and Raichle, 2012; Power et al., 2012) may be not very adjuvant and even counterproductive. To avoid the suppression of any meaningful variance around 0.1 Hz only SPM standard realignment (rigid body transformation) was performed for all subjects. We believe that there is no reason to suppose that motion artefacts would express themselves differently in these subgroups and therefore act as confounds for the inter-group comparisons carried out by us.

1. Head motion
It has been established that head motion is one of the major sources of artefacts in all MRI brain studies, especially with fMRI and functional connectivity (see Power et al 2012). The authors are not mentioning if and how motion correction was performed during preprocessing. The following issues should be resolved:

Power et al. (2012) demonstrated in their excellent paper that BOLD signals in the frequency band 0.009–0.08 Hz are sensitive to subject motion and need therefore methods to reduce motion-related effects. In our case of studying BOLD signals in the frequency band 0.07–0.13 Hz between subgroups there is no reason to suppose that head motion effects would express themselves differently in these subgroups.

The authors should report if motion correction was performed

SPM standard realignment (rigid body transformation) was performed for all subjects.

The authors should report the amount of framewise displacement (FD) as percentage of volumes with FD above 0.2 mm for each subject/session

FD was not recorded.

The authors should report if they used techniques to mitigate head motion artefacts such as one of these three: i) regression of head motion parameters, ii) ICA-AROMA, iii) FSL FIX, or other similar procedures.

None of these techniques was used. We decided to abstain from applying standard motion artefact techniques described in the literature, because A) we wanted to avoid suppressing meaningful variance around 0.1 Hz and B) our concern was differential connectivity patterns between subgroups (e.g. neural vs vascular oscillations), and there is no reason to suppose that head motion artefacts would express themselves differently in these subgroups. The goal of standard techniques of motion correction is to suppress all non-neural activity. Vascular BOLD oscillations belong also to non-neural signal, but are subject of our PLV analyses.

If no technique for reducing the impact of motion artefact was in place, the authors should re-run preprocessing, possibly using ICA-AROMA as it seems to be the best performer

Re-running the preprocessing stage would be logistically unfeasible and we do not feel that it would be appropriate, for the reasons outlined above.

Other techniques can be used to mitigate the effect of head motion such as scrubbing (censoring time points with FD > 0.2mm) or removal of global signal. Was any scrubbing in place? The authors should also explicitly report if global signal was removed or not.

No scrubbing was applied. No global signal correction was applied. We opted to avoid the application of motion correction techniques which have been tested and validated in other contexts but that we feel would be detrimental in a case where the goal was to compare groups using resting-state data rather that assessing statistically relevant, task-induced or resting-state effects in homogeneous populations which might be potentially confounded by systematic head motion effects.

Related to the previous point: the authors could clarify how many time points were used in estimating the PLV, given also the fact that with wavelet transform, the cone of influence greatly limits the total amount of time-points that are free from artefact (as noted also by the authors in the manuscript). The authors only mentioned 40s per time point, but they could specify the total number of time points and the amount of time point removed at the beginning and at the end due to the COI of the wavelet transform.

A total of 32 points (16 in the beginning and 16 in the end of the time series) were removed due to cone of influence.

2 Computing average PLV
The authors mention that "averaging was restricted to values for which PLV was significant", which can leave the reader wonder whether this is a source of bias, what was happening at the time points when PLV was not significant, and why they were discarded a priori. The author should first test if the instantaneous p-value of PLV correlates with instantaneous FD, i.e. to make sure that p-values (or better PLV effect sizes) are not co-varying with head motion.
Furthermore, I would suggest to use all PLV values and rather than averaging the strongest ones, the whole distribution of PLV values can be used as fingerprint of each specific scanning session. Then multivariate test comparing distributions could then separate subjects into two groups as mentioned later in the manuscript. However, this is not necessary for this manuscript, although the authors should acknowledge in the discussion that there is a potential source of bias in considering only strongest PLV values.

Phase difference is very unstable (by definition) outside segments where PLV is high. Therefore, we opted to focus on segments where the null hypothesis of no phase-locking could be rejected, and therefore the estimates of phase difference (and hence time delay values) were more reliable. From a physiological point of view, it is plausible to suppose that signals fluctuate between epochs of high and low phase-locking. The latter are more meaningful because they correspond to periods where effective inter-regional communication is taking place.

3 When considering $p<0.05$ in the selection of PLV time points, the authors are not controlling for the multiple comparisons. I.e. the alpha should be $0.05/T$ with T the total number of time points considered. However, as this Bonferroni approach does not take into account the autocorrelation of the signal, the authors could estimate the intrinsic degrees of freedom of PLV time series and correct for those i.e. setting the alpha at $0.05/DF$ (if the PLV signal was white Gaussian then $DF \sim T$; from experience, I expect $DF \sim T/10$ or smaller).

That would indeed be a way to handle multiple comparisons. However, we didn't feel it to be necessary in our case. We are not trying to verify a hypothesis concerning the number of significant time points based on a PLV threshold. The 0.05 threshold is a tool to select segments for stable computation of mean PLV (and hence time delay), and it is quite arbitrary. Any kind of multiple comparison correction would reduce the amount of available time points for mean PLV computation and would not be statistically relevant for our purposes.

4 Results in Figure 2
It is an expected result to obtain a p-value of E-10 when comparing the time delays of the two groups, as the two groups were indeed formed based on their time delays. I personally wouldn't stress the "extreme large effect size". This might also be a consequence of using only the strongest (PLV p-value < 0.05) when computing the averages. The result would have been more interesting with average PLV across all time points. However, it is not clear if these pTD and nTD values used for testing are indeed the average PLV over the $p < 0.05$ time points. Could the author please clarify how pTD and nTD were calculated? Furthermore, if they are in seconds, the unit measure should be added to the y axis in Figure 2.

Thank you for this helpful comment. We agree that the strong emphasis on the "extremely large effect size" is somewhat redundant and toned down the language accordingly.
Please note that all results are based on PLV values computed using only the points above the $p<0.05$ threshold.

5 Figure 2: it is redundant to have both A, B, C and D, E, F charts as they convey the same information. Mean and quartiles can be added to the panel A, B, C (e.g. by overlaying a box plot or a violin plot) and the barplots can be removed.

We agree on the redundancy of both graph types and replaced them with boxplots.

6 Figure 2: as the same individual is in both group A and group B, the dots from the same individual should be connected with a line to show a possible interaction. This is necessary when plotting paired data, see Figure 2 in Tracey et al 2015

Due to the use of boxplots we abstained from using lined stripplots. However, should the reviewer insist on this, we would certainly be willing to comply.AS

7 Figure 2: a similar one-sample t-test could be done also using the mean framewise displacement of each session with pTD vs nTD to show that average motion did not correlate with pTD and nTD.

FD was not recorded (see above).

8 Results of Figure 4: there is no mention of multiple comparisons correction. Specifically, since there are 26 ROIs, the total number of comparisons are 26(26-1)/2=325. False discovery rate correction could be used or, even better, the Network Based Statistics method for controlling FDR when comparing link weights.*

Multiple comparison correction as available in the GraphVar package (Kruschwitz J D, et al. (2015) GraphVar: a user-friendly toolbox for comprehensive graph analyses of functional brain connectivity. J Neurosci Methods 245:107-115) was applied in this case.

9 The reader is left wondering if a standard method for computing functional connectivity between the 26 ROIs would have provided similar results. Since most of connectivity literature is based on Pearson's correlation of 0.01-0.1 Hz BOLD time series, the authors should compute a figure similar to Figure 4 using standard connectivity procedures between the same groups: group A and group B. This is interesting independently on the direction of the result: if the PLV 0.1 Hz result overlaps with standard functional connectivity techniques, the authors can further comment on the neuronal contribution of 0.1 Hz oscillations. On the other hand, if the two methods give different results, it could be that PLV is showing something more (e.g. related to Alpha and Beta ERD as seen in Figure 3 since low frequency BOLD contribution is mainly reflected at the Gamma band, cf. Logothetis' work).

An increase of neural activity is generally associated with a decrease of alpha and/or beta power. This would indeed be a meaningful improvement. However, in this case we were specifically focusing on the phase component because it was more meaningful to our hypothesis. Standard measures such as Pearson's correlation and magnitude square coherence are both unable to untangle phase and magnitude effects. Thank you for mentioning the important work of Logothetis. We modified the legend of Fig. 3: "An increase of neural activity is generally associated with a decrease of alpha and/or beta power *(ERD; Pfurtscheller & Lopes da Silva, 1999) and a gamma increase (Logothetis and Wandell, 2004)."*

10 Figure 5: same considerations as in, Figure 2 above (remove bar plots, overlay a box plot or violin plot if necessary or just the average, connect paired data points with a line)

We agree on the redundancy of both graph types and replaced them with boxplots.

11 The manuscript is lacking a "limitations" paragraph in the discussion section. The authors could mention at least these four limitations:

EJN European Journal of Neuroscience FENS Federation of European Neuroscience Societies

(i the authors do not discuss breathing as a possible driving factor in the neural/vascular BOLD separation. For example, the unexplained 0.17 Hz component in Figure 6 could correspond to a breathing rate of 10 breath/min which would not be too unusual during rest. Furthermore, breathing and chest movements during breathing introduce systematic head motion as well as inhomogeneity of the scanner magnetic field. Finally breathing rate is related to heart rate through the vagal tone, hence we cannot exclude that what we see here is related to heart-breathing synchronization reflected in the brain. Please refer to these two links for many references on these topics: 1] https://practicalfmri.blogspot.fi/2017/08/fluctuations-and-biases-in-fmri-data.html 2] https://practicalfmri.blogspot.fi/2014/12/concomitant-physiological-changes-as.html

We like to thank the Reviewer for these important references but it should be mentioned that in Fig. 1 with the major routes in time series data in an fMRI experiment (Krainik et al. 2013) one important route is not explicit mentioned, namely the descending “central commands/ central pacemaker” route (Julian 2006) responsible for the fast (vagal nerve) rhythmic modulation of the HRV in the low frequency band (0.1 Hz).

ii) The size of each ROI is different with amygdala having lower amount of voxels than cingulum, hence the “less smooth” time series in Fig 6. (At 2mm istropic, AAL 33 “Cingulum_Mid_L” has 1943 voxels, while AAL 41 “Amygdala_L” has 234). In general, ROI size constitutes a source of artefact as the bigger the ROI the less consistent the ROI signal is
iii) As the population was not normative, it should be stressed that these results might not extend to the healthy population.

The population studied was a group of healthy individuals (students) without former experience with a scanner experiment (scanner-naïve subjects)

iv) As the Nyquist frequency for the BOLD is around 0.5 Hz, there could still be aliasing due to the heart signal introducing distortion in the observed frequency band. The reader is left wondering what would have been the results if methods such as RETROICOR were used.

In the new paragraph “Limitations and future prospects” the comments mentioned in point 11 (i), (ii) and (iv) were discussed.

Minor comments
- The authors did not mention the number of channels in the coil used, I assume 32 since the MB sequence used is somewhat similar to that used by the Human Connectome Project.

For data acquisition we used the 32-channel head coil and a multiband GE-EPI sequence (Moeller et al. 2010). We added this information in the paragraph **fMRI and phase-locking calculation** of the revised manuscript.

- The authors did not mention the type of head-restraint use (I presume foam padding)

Yes, we used foam pads to minimize the head motion. We added this information on page 5 of the revised manuscript.

- The authors did not specify what type of methods was use to compensate for scanner drift (I presume linear or spline detrending)

We used linear detrending. We added this information in the paragraph **fMRI and phase-locking calculation** of the revised manuscript.

- I have spotted one typo (Golanow should be spelled Golanov)

Thank you, corrected.

EJN European Journal of Neuroscience FENS Federation of European Neuroscience Societies

Reviewer 2

In the whole manuscript, I could not find the description about statistics, what kind of statistic methods, which software.

More complete information about these topics has been added to the manuscript.

Page 4 and Page 5 about preprocessing of fMRI data, why no relignment step? why used 3 x 3 x 3 mm to normalization?

SPM standard realignment (rigid body transformation) was performed for all subjects. This is what was meant by “motion correction”.
3x3x3 mm resampling is a default option in the Normalization step of DPARSF. We assume it is related to the spatial resolution of the template that DPARSF uses.

Table 1 should be modified.

We modified Table 1 accordingly and hope that it is now better digestible.

3rd Editorial Decision 05 December 2017

Dear Dr. Brunner,

Your revised manuscript was re-evaluated by one of our original external reviewers as well as by the editors. Although your manuscript has been substantially improved, there continue to be a few relatively minor concerns that need to be addressed more effectively before we can proceed to publication. As you will read below, the reviewer calls for some additional objective measures of potential motion artefacts and we strongly encourage you to execute the proposed measurements and to document them in the paper itself. They should be easily done with the provided code.

Also, please attend to the following issues identified by our editorial office:
1. At this stage, please provide text and a figure file for the Graphical Abstract.
2. Your figure captions should be in a list at the end of the manuscript, not embedded in the text
3. Your figures are still rather small and low resolution, so please provide much better versions
4. Your tables are embedded and will need to be moved to end of manuscript

If you are able to respond fully to the points raised, we shall be pleased to receive a revision of your paper within 30 days.

Thank you for submitting your work to EJN.

Kind regards,

John Foxe & Paul Bolam
co-Editors in Chief, EJN

Reviews:

Reviewer: 1 (Enrico Glerean, Aalto University, Finland)

Comments to the Author
I would like to thank the authors for following my suggestions in this new revised version of the manuscript. I think most of the issues were well covered. I agree with them that

"The investigation of neural vs. non-neural BOLD oscillations in the low frequency band at 0.1 Hz is a novel approach and therefore the standard procedures to correct for non-neural BOLD signals (Fox and Raichle, 2007; Snyder and Raichle, 2012; Power et al., 2012) may be not very adjuvant and even counterproductive.

To avoid the suppression of any meaningful variance around 0.1 Hz only SPM standard realignment (rigid body transformation) was performed for all subjects."

A similar statement was included in the limitations and I think a similar shorter version could also go in the methods section since many other scientists will be left wondering why you did not use tools for mitigating head motion effects.

However, it is anyway good practice to report a quality control measure of the amount of head motion even if you do not expect it to affect your results (back in the days it was usually reported in mm with 2mm considered high quality data, now it is often reported using framewise displacement).

Furthermore, the statement:

"We believe that there is no reason to suppose that motion artefacts would express themselves differently in these subgroups and therefore act as confounds for the inter-group comparisons carried out by us."

can be easily tested with your data.

For this reason, I would like the authors to compute framewise displacement (FD) time series (it is very easy to compute them from the SPM motion parameters, see an example Matlab function that I wrote: https://version.aalto.fi/gitlab/BML/bramila/blob/master/bramila_framewiseDisplacement.m)

With FD time series the authors can then compute i) the percentage of volumes above 0.5 (used in Power et al 2012) to be reported as quality control measure and ii) the average FD across each of the resting state runs.

The authors can then use the mean FD for each subjects in group A and B in figures 2 and 5 (i.e. the two boxplot figures) to show that the groups are matched in the amount of head motion. A two sample t-test using each subject's mean FD can then be reported for figure 2 and 5. If it turns out that there is significant difference, then a linear model using mean FD as covariate of no interest can be used to test group A vs group B.

Minor comment:
I would like to thank the authors for considering my suggestion of introducing a limitations paragraph at the end of the discussion. I just want to point out that the link between the first and second sentence is somewhat broken. The first sentence is about the Nyquist frequency and spectral aliasing (corrupting the highest frequencies) while the second sentence is about head motion. So the "This" at the beginning of the second sentence should be about head motion, not about spectral aliasing.

best
Enrico Glerean

Authors' Response 16 January 2018

Thank you very much for your decision letter of December 5th. We performed the requested framewise displacement (FD) analysis and confirmed that there is no significant difference between the two groups. Enclosed please find the revised submission (changes are underlined), also including figures with better quality.

Reviewer 1

I would like to thank the authors for following my suggestions in this new revised version of the manuscript. I think most of the issues were well covered. I agree with them that "The investigation of neural vs. non-neural BOLD oscillations in the low frequency band at 0.1 Hz is a novel approach and therefore the standard procedures to correct for non-neural BOLD signals (Fox and Raichle, 2007; Snyder and Raichle, 2012; Power et al., 2012) may be not very adjuvant and even counterproductive. To avoid the suppression of any meaningful variance around 0.1 Hz only SPM standard realignment (rigid body transformation) was performed for all subjects."

EJN European Journal of Neuroscience FENS Federation of European Neuroscience Societies

A similar statement was included in the limitations and I think a similar shorter version could also go in the methods section since many other scientists will be left wondering why you did not use tools for mitigating head motion effects.

The following sentences were inserted in the Method section:

To avoid the suppression of any meaningful variance around 0.1 Hz, only SPM standard realignment (rigid body transformation) was performed for all subjects. Standard procedures to correct for non-neural BOLD signals (Fox et al., 2007; Snyder and Raichle, 2012; Power et al., 2012) were skipped because they were deemed to be potentially counter-productive in this context. Only the framewise displacement (FD) (Power et al., 2012) was computed in order to rule out that any subsequent results might be confounded by motion artifacts.

For this reason, I would like the authors to compute framewise displacement (FD) time series (it is very easy to compute them from the SPM motion parameters, see an example Matlab function that I wrote: https://version.aalto.fi/gitlab/BML/bramila/blob/master/bramila_framewiseDisplacement.m)

With FD time series the authors can then compute i) the percentage of volumes above 0.5 (used in Power et al 2012) to be reported as quality control measure and ii) the average FD across each of the resting state runs.

The authors can then use the mean FD for each subjects in group A and B in figures 2 and 5 (i.e. the two boxplot figures) to show that the groups are matched in the amount of head motion. A two sample t-test using each subject's mean FD can then be reported for figure 2 and 5. If it turns out that there is significant difference, then a linear model using mean FD as covariate of no interest can be used to test group A vs group B.

Thank you for this suggestion. We computed mean FD in both groups and confirmed that there is no significant difference. The following sentence was inserted:

The comparison of the framewise displacement (FD) in both groups is illustrated in Fig. 2D and revealed no difference (pTD: M = 0.17 mm ± 0.07; nTD: M = 0.17 mm ± 0.11); $t(17) = 0.013$, $p = 0.99$).

I would like to thank the authors for considering my suggestion of introducing a limitations paragraph at the end of the discussion. I just want to point out that the link between the first and second sentence is somewhat broken. The first sentence is about the Nyquist frequency and spectral aliasing (corrupting the highest frequencies) while the second sentence is about head motion. So the "This" at the beginning of the second sentence should be about head motion, not about spectral aliasing.

Thank you, we clarified this sentence:

Head motion is definitely a problem if the connectivity between BOLD signals is studied below 0.1 Hz, but is of less importance by analyzing a narrow frequency band around 0.1 Hz in BOLD and RRI signals.
